# Thermodynamic consequences of the kinetic nature of the glass transition

**DOI:** 10.1038/srep17782

**Published:** 2015-12-10

**Authors:** Kajetan Koperwas, Andrzej Grzybowski, Satya N. Tripathy, Elzbieta Masiewicz, Marian Paluch

**Affiliations:** 1 Institute of Physics, University of Silesia, Uniwersytecka 4, 40-007 Katowice, Poland; 2 Silesian Center for Education and Interdisciplinary Research, 75 Pulku Piechoty 1A, 41-500 Chorzow, Poland

## Abstract

In this paper, we consider the glass transition as a kinetic process and establish one universal equation for the pressure coefficient of the glass transition temperature, dT_g_/dp, which is a thermodynamic characteristic of this process. Our findings challenge the common previous expectations concerning key characteristics of the transformation from the liquid to the glassy state, because it suggests that without employing an additional condition, met in the glass transition, derivation of the two independent equations for dT_g_/dp is not possible. Hence, the relation among the thermodynamic coefficients, which could be equivalent to the well-known Prigogine-Defay ratio for the process under consideration, cannot be obtained. Besides, by comparing the predictions of our universal equation for dT_g_/dp and Ehrenfest equations, we find the aforementioned supplementary restriction, which must be met to use the Prigogine-Defay ratio for the glass transition.

The fundamental mechanism underlying the glass transition phenomena in non-crystallizing liquids is perhaps the most challenging problems in condensed matter physics and active areas of research since 1950. In the quest to deliver a complete explanation of the transformation from metastable supercooled state to the non-equilibrium glassy state, abundant theoretical and experimental studies have been devoted. Particularly, the change in glass transition temperature (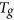) as a function of pressure and its connection with the thermodynamic coefficients, which provides a suitable parameter to elucidate the nature of glass transition, has been intensively examined. Numerous experimental studies on different glass-formers reveal that the pressure coefficient of the glass transition temperature is substantial in the case van der Waals liquids (*i.e.*, 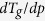 ≈ 0.25 K/MPa)^1^,^2^, whereas for hydrogen-bonded liquids the change of 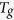 weakly depends on pressure (*i.e.*, 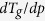 ≈ 0.1 K/MPa)^3^,^4^. Certainly, the development of a suitable universal relation between 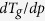 and the key thermodynamic coefficients (*i.e.*, isobaric expansivity (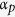), isothermal compressibility (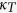), isobaric (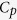) and isochoric (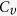) specific heats) has become a fundamental problem of the theoretical investigation of glass physics.

In the past, many efforts have been gained to explain the nature of the glass transition. It is well established, that at the glass transition, Gibbs free energy and its first derivatives (*i.e.*, volume 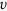 and entropy 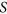) are continuous, while second derivatives are connected to thermodynamic coefficients and show step-like behavior in the vicinity of 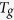. Eventually, attempts have been made to consider the liquid-glass transition as a second-order phase transition. Some efforts were undertaken to verify both Ehrenfest equations (eqs. (1) and (2)) and the Prigogine-Defay ratio (eq. (3))^5^,^6^,^7^.




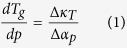







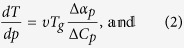







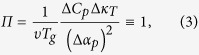




where 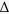 denotes the differences between respective coefficients in the liquid and the glassy states. However, it is worth noting that in the case of a second-order phase transition the system goes from one equilibrium state to the other, whereas at the glass transition, the system is transferred from the (metastable) equilibrium into the non-equilibrium state, which is the glass. Therefore, it is not surprising that the majority of experimental investigations of “
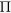
” revealed that its value differs from unity^5^,^8^,^9^,^10^. Another approach to describe the liquid-glass transition, which is certainly worthy of attention, is the concept of order parameters, which was introduced by Donder and van Rysselberghe^11^. It suggests that the state of the system in both the equilibrium and non-equilibrium states depends on the thermodynamic intensive parameters (temperature, pressure) and a number of order parameters. Then, Simon proposed that the glass transition could be considered as a process at which the system becomes suddenly kinetically frozen in^12^, *i.e*., the structural reorganization cannot follow anymore the change of temperature and/or pressure. Assuming that only one order parameter is sufficient to describe the structural differences between the liquid and the glass, both Ehrenfest equations and hence value 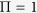 were theoretically obtained by Davies and Jones 5,10,13,14. In this context, it has to be noted that equation (1), which is usually not fulfilled^14^,^15^,^16^,^17^,^18^, incorporates 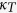 also measured in the nonequilibrium glassy state, which makes it difficult to verify experimentally^19^. On the other hand, equation (2) seems to hold reasonably well for many systems, although not for all^20^,^21^. Nevertheless, the Prigogine-Defay ratio is seen as an indicator of the complexity; the number suggesting the degree to which the liquid fails to be described by a single order parameter^22^. However, treating vitrification in terms of Simon’s models^12^, one assumes that the order parameter is defined independently on the rate of external parameters changes as a function of temperature and pressure, which in general is not true, because for dense systems during the cooling to 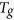 and below the order parameter cannot follow the changes of the external parameters (*e.g.*, temperature) and deviates from its equilibrium value^23^. This behavior occurs when the time scale of changes of the external parameter become comparable with the characteristic relaxation time of the system 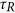 *e.g.*, the structural relaxation time 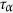. Therefore, the transformation from the liquid to the glassy state is expected to have a kinetic, rather than thermodynamic, origin. It is consistent with the one of the most important conclusions from the long history of the research on the glass transition process, i.e., the dependence of 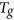 on the experiment (cooling or heating) rate. Hence, many efforts have been made to formulate the definition of the glass transition by considering it as a purely kinetic process. First attempts were made by Bartenev^24^ and Jones^25^, in 1949. Two years later, from a general examination of the cooling process^24^,^26^, Bartenev derived the relation 
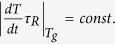
24, which was experimentally corroborated for different materials^27^,^28^. It is worth noting that an identical formula can be obtained from the chemical reaction model when one employs certain additional conditions, which are commonly met in the glass transition, as Volkenstein and Ptizyn presented^24^. They also noticed that the constant term occurs in the Bartenev’s relation should weakly depend on 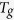. Nevertheless, later and detailed examinations performed by Moynihan *et al.*^29^ have confirmed that the aforementioned relationship is perfectly satisfied, and then it has become regarded as generally binding at the glass transition^30^,^31^. Thereby, the general understanding has been shaped, according to which, only the value of 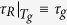 defines the glass transition, if the constant cooling rate is applied to different isobaric states. In this context, the interesting considerations given by Hodge^32^, to the Deborah number, which is defined as the ratio of timescales of the observed and the observer, 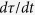, are worthy of mentioning. According to them the glass transition is seen when the above two timescales cross over and then Deborah number passes through unity. Then in the temperature domain, that is explored most thoroughly, a Deborah number of unity that defines an average 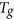 can be expressed in terms of the rate of change of some characteristic timescale 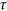, determined during cooling: 
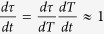
. Since the temperature dependence of relaxation time for many processes is given by the empirical Vogel-Tamman-Fulcher equation, at the isobaric conditions the above criterion results in the following relation 
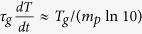
, where 
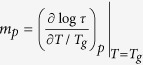
is the isobaric fragility. Assuming that the glass transition is approached at a constant cooling rate, 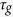 becomes proportional to 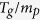. Then, combining it with Bartenev relation, one obtains that the constant value depends on 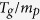, which implies that values of 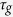 do not have to be invariant for all isobaric conditions. However, 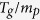
established by experimental data of the structural relaxation times 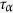, obtained from dielectric spectroscopy of different glass formers, is a slowly varying function of the pressure, which usually results in the increase in 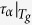 in only a few seconds with increasing the pressure from 0 to 200 to 300 MPa^33^. Hence, the glass transition can be defined, with very good agreement, by a constant value of 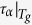
(which for simplicity in the later part will be denoted by 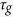). Following the general wisdom, in this paper we examine the glass transition as a kinetic process and consequently on this basis, we derive one universal equation for the pressure coefficient of the glass transition temperature. We show that by considering the dependence of the characteristic relaxation of the system, defined by the structural relaxation time 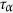, on different external parameters, one can obtain equations for 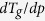, which originates from one universal equation. In this context, they are not independent, and hence the relation equivalent to the Prigogine-Defay ratio cannot be established for the glass transition, at least without employment of an additional condition met in this process. The three above possible equations for 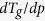, obtained in the cases of dependences, 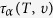, 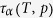, and 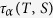, have been verified. We also show that predictions based on the above equations are consistent within a wide range of pressures. Moreover, we analyze our universal equations in terms of the Simon’s model^12^ and we find for which systems the structural differences between the liquid and the glass can be described by only one order parameter.

## Results

**Figure 1:**
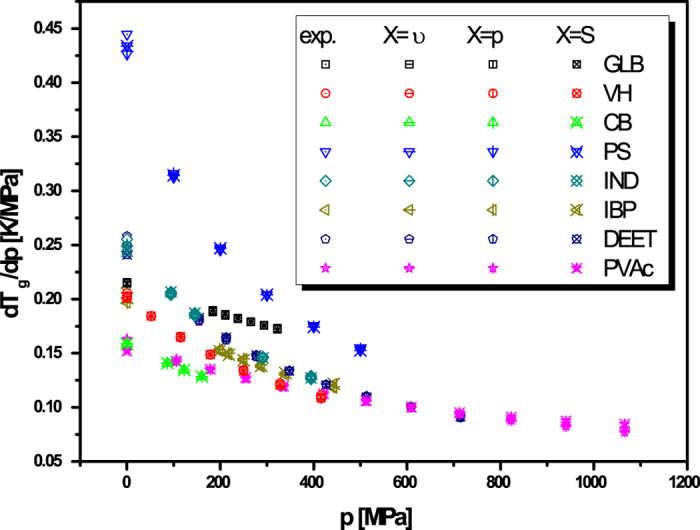
Values of the pressure coefficient of the glass transition temperature calculated from equations (5, 6, 7), 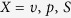 respectively, and obtained from analysis of experimental measurements (exp.) for glibenclamide (GLB), verapamil hydrochlorine (VH), carvedilol base (CB), polystyrene (PS) (all in Ref. 33), indometacin (IND)^59^, ibuprofen (IBP)^60^, N, N-dimethyl-3-methylbenzamide (DEET), and polyvinylacetate (PVAc). The glass transition for CB is defined by 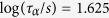, whereas for DEET and PVAc by 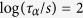.

The structural relaxation time 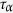 depends on many thermodynamic quantities, *e.g.*, temperature, pressure, volume, and entropy. However, only two of them can be changed independently from the others. Since temperature is the physical quantity most often controlled in different experiments, we propose that in the most convenient way, the structural relaxation time can be considered as a function of 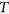 and another thermodynamic quantity 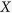. Then, the complete differential of the structural relaxation time 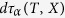, at the glass transition defined at a constant value of 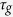, equals 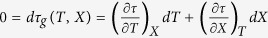. On the other hand, 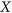 is a function of two independent thermodynamic quantities that can be selected from many of the other thermodynamic quantities. One of the most natural selections is 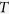 and 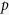, and then 
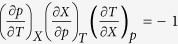
. Using the above relation between thermodynamic quantities we can rewrite the complete derivative of 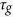 and then 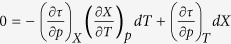. Since, 
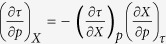
, the latter equation transforms to 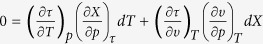, where expressions for the isobaric and the isothermal fragility (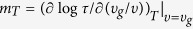) can be exploited. As we have mentioned above, 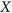 is a function of 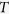 and 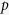, thus the formula for its complete derivative is expressed as follows 
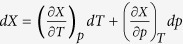
. Using it, we obtain the required equation for the pressure coefficient of the glass transition temperature, which takes the subsequent form 
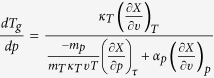
. The term, which appears in the denominator and consists of quotient of the isobaric and the isothermal fragility, can be transformed to 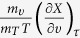, where 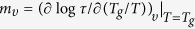 is the isochoric fragility. In conclusion, the general equation for the pressure coefficient of the glass transition temperature takes the following form




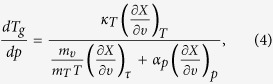




where 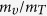 reflects the relative roles of 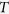 and 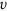 in the molecular dynamics, whereas 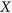 is any thermodynamic quantity, *e.g.*, 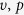 or 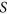. Thus, one can easily obtain several expressions for 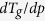, *e.g.*,




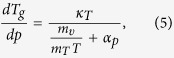







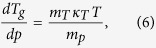







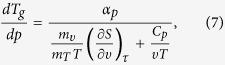




if one substitutes 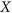 for volume (eq. (5)), pressure (eq. (6)), entropy (eq. (7)) or another physical quantity. The predictions of above equations for 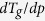 and values of this coefficient obtained from analysis of the experimental measurements are presented in Fig. 1, which shows that the values of 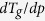 calculated from equations (5), (6), and (7) are very consistent with each other over a wide range of pressures and as well as to that received from the analysis of the experimental data. In this context, it has to be noted that equation (5) was earlier successfully verified at ambient pressure for many glass-forming liquids that belong to the different material groups^33^.

It is worth noting that the universal character of the equivalent equations (5)–(7), can be extended beyond the assumed case of the dependence 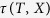. Considering the density scaling laws for 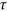 and 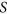
(discussed later for 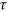 and in the Supplementary Information for 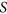), we have very recently argued^34^ that the structural relaxation time can be in general a volume-entropy function 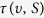. Then, equation (5) also remains valid as shown in Supplementary Information.

## Discussion

The excellent agreement between values of 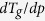 predicted by equations (5)–(7), is not surprise since all equations are received from equation (4). It has to be mentioned that employing the relation between fragilities 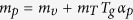
35; one can easily transform equation (6) to equation (5) as well as by using the thermodynamic relation 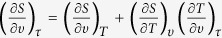, equation (7) can be converted to equation (5). Taking the above into account, we can expect that knowledge of the relationship between any 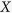 and 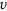 enables transformation of whichever equation for 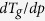 resulted from equation (4) to equation (5). Besides, derivation of two independent equations for 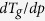, and an establishment of the relation among the thermodynamic coefficients at the glass transition (corresponding to the Prigogine-Defay ratio), is not possible, at least based on only the kinetic description of this process. So, in order to establish the relation equivalent to equation (3) that is satisfied at the glass transition, we must employ an additional condition met in this process. This has been done in Ref. 36 in which Eq. (5), which is the exceptional form of equation (5) (derived from the density scaling law, what will be discussed later), is connected with one of the Ehrenfest equations. In the next paragraph we present the derivation of the Ehrenfest equations from our universal equation for the pressure coefficient of the glass transition temperature. However, now we can mention that this procedure requires additional assumptions and hence equation (5) from ref. 36 cannot be simply transformed to one of Ehrenfest equations. Therefore, the main equation in the mentioned paper, i.e., equation (7), results from the connection of two independent equations and is not the tautology.

In this paragraph we consider the universal equation for the pressure coefficient of the glass transition temperature in terms of the Simon’s model employed by Davies and Jones^10^, to give a new look at the system for which the Prigogine-Defay ratio should be valid, *i.e.*, single order parameter systems. According to simplification of the Simon’s model used by Davies and Jones, the glass transition takes place at a singular 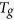, at which the order parameter became kinetically frozen-it. Then, 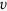 and 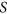 of the metastable liquid (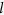) and the glass (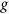) have the same values, which implies that the configurational volume 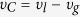 and entropy 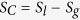 equal 0. Since, 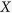 introduced by us in equation (4), can be any thermodynamic quantity, there is nothing to preclude the consideration dependence of the characteristic relaxation time of the system on configurational values e.q. 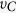 or 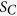. In this way, equation (4) takes following forms




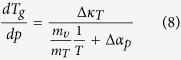







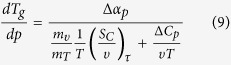




for 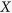 given by 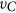 and 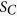 respectively. Now, it can be seen that equations (8) and (9) will become identical with equations (1) and (2) if the quotient of isochoric and isothermal fragility equals 0, which implies that 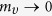 or 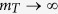. Both the restrictions reflect one possible but limited behavior of the molecular dynamics, *i.e.*, the situation at which molecular dynamics is controlled only by local density fluctuations. Thereby, the so-called “free volume model” is expected to be suitable for single order parameter liquids. Moreover, it has to be noted that the Ehrenfest equations are limiting cases of our universal equation for 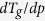, which seems to be much more general. On the other hand, if only fluctuations of the temperature govern the molecular dynamics, 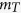 and 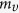 behave conversely to before, and hence 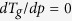, which indeed reflects the situation at which the glass transition occurs at constant temperature independently of pressure and hence volume.

An additional finding, which advances our knowledge of the single order parameter systems, can be deduced from the density scaling idea. It is worth mentioned that alternative study of the so-called linear Prigogine-Defay ratio in the context of the density scaling has been very recently performed^37^. Density scaling postulates that the relaxation time of the system can be expressed by only one variable as follows, 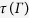, where 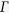 is a function of 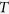 and 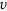. In the most common form, which has been experimentally validated for more then 100 van der Waals liquids and polymers, 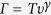
38,39,40,41 where 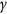 is a scaling exponent, which is only material dependent. One of the consequences of the above form of the density scaling is a potential connection between 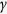 and quotient of isochoric and isothermal fragility, 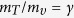
35. Then, equations (5) and (6) can be expressed in the following form 
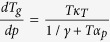
, whereas equation (7) transforms to 
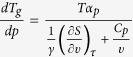
. Both new variants of our universal equation for 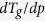 deserve special attention because remarkably, many computer simulations of molecular dynamics confirm the connection between 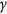 and an effective exponent used to model the repulsive part of intermolecular potential^42^,^43^,^44^,^45^,^46^,^47^. For dense systems, the physically relevant intermolecular potential can be successfully approximated by an effective IPL potential consisted of a dominating Inverse Power Law term for repulsive interactions (proportional to the power of intermolecular distance 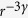) and small nearly constant background reflecting attractive forces. Taking into account the previously mentioned issue of the “free volume model”, systems which molecular dynamics is controlled only by the density fluctuations, may be modeled by the effective IPL potential, where the power of intermolecular distance tends to infinity, 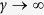. Therefore, they become similar to the hard sphere systems with small and constant background reflecting attractive forces. So, we can expect that the Prigogine-Defay ratio describes the relation among thermodynamic coefficients at the glass transition for systems modeled, *e.g.*, by the hard sphere potential. However, the formulation of the precise definition of the order parameter, which became kinetically frozen-in at the glass transition, remains an open issue in glassy physics.

At the end, we provide a comment about the excellent work of J.W.P. Schmelzer^7^, in which author introduced the new general criterion for the glass transition, which consider the cooling or heating rates, 
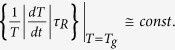
, where 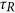 is a characteristic relaxation time of the system. On the basis of the above criterion, the author derived the equation for 
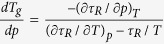
 (equation (41) in ref. 7). Later, Schmelzer examined his equation in the limiting case of the “free volume model” and the “entropy-based approach”. As a result, he obtained 
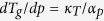
 for the “free volume systems” as well as 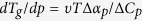, which is one of the Ehrenfest equations (eq. (2)), for the “entropy-based approach”, *i.e.*, considering the relaxation times as a quantity, which depends on the temperature and the activation energy determined by the entropy (respectively equations (46) and (50) in ref. 7). It has to be noted that our universal equation for the pressure coefficient of the glass transition temperature (eq. (4)) considered in terms of the temperature-pressure dependence of the relaxation time (eq. (5)) and the “free volume model” provides an expression for 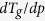, which is identical with that derived by Schmelzer for these limiting cases under consideration. However, a more intriguing fact is that Schmelzer established one of the Ehrenfest equations employing only the “entropy-based approach”, because this approach to describe the molecular dynamics, is not dedicated only to the systems which dynamics is governed solely by the local volume fluctuations. This finding is in contrast to our result since in the previous paragraph we noticed that the use of “free volume model” is necessary to obtain the Ehrenfest equations from our universal equation for 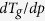. The only assumption made by Schmeltzer during his analysis is 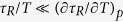, which results that 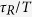 can be neglected. It has to be mentioned that the author emphasizes that the validity of the above restriction is limited. However, we want to show that using the following thermodynamic relation 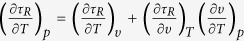; one can rewrite the denominator under consideration and then 
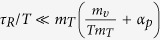
, which is fulfilled independently of 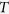 and 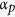 when 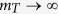. Thus, Schmeltzer obliviously limited his analysis to the “free volume model”, and hence the “entropy based approach” employed by the author is, in fact, suitable only for the system, the dynamics of which is controlled purely by the fluctuations of volume. The presence of 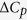 and 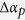 in the expression derived by Schmeltzer result from the entropy model used by him, *i.e.*, Adam-Gibbs model, which considers an influence of the configurational value of the entropy on the relaxation time. It is also worth noting that the term 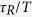 results from the consideration of experiment (cooling or heating) rate in Schmelzer’s general criterion for the glass transition, thus its omitting, which is caused by the assumption that 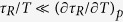, limits the predictions of the above criterion to conditions at which the glass transition is characterized by a constant value of characteristic relaxation time. In this way, the results obtained by Schmelzer are consistent in a whole with our universal equation for 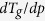. However, Schmelzer’s work draws our attention to the consideration of cooling or heating rate as a promising opportunity to establish of another sought after condition met in the glass transition. Unfortunately, the general criterion proposed by the author seems to be not suitable for real materials, because one obtains that 
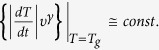
 after considering it in terms of the density scaling, 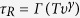, and then for a constant value of 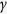, the glass transition should takes place at a constant volume in varying thermodynamic conditions (T and P), which is not true. Therefore, another form of the general criterion for the glass transition should be found, if it is possible.

In summary, we have derived a new general equation for the pressure coefficient of the glass transition temperature, equation (4), based on the kinetic definition of the process was experimentally verified. The ultimate advantage of our new equation is its universality, *i.e.*, it does not depend on the physical quantities, which describe the dependence of the relaxation time of the system. The consequence of the existence of one universal equation for 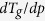 is the fact that the derivation of the two independent equations for 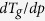 is not possible, at least without use of an additional condition, met in the glass transition. Our finding suggests that the relation equivalent to the Prigogine-Defay ratio, which results from a combination of the two independent equations for 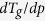, might not exist at the glass transition. For an example, we present three different equations for the pressure coefficient of the glass transition temperature, equations (5), (6), and (7), from which, anyone can be transformed to another. It should be mentioned that the above conclusion are proper for any process, which occurs at isochronal conditions, e.g., for nematic-isotropic transition^48^,^49^,^50^,^51^ or smecticE-isotropic transition^52^ observed in liquid crystals. Moreover, we show that the well-known Ehrenfest equations can be derived from our universal equation for 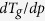, when terms of the Simon’s model are employed, as Davies and Jones did when they obtained the expressions for 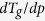 suitable for the single order parameter systems, *i.e.*, Ehrenfest equations. Since a possibility of use of our universal equation for 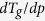 does not depend on the number of order parameters, we deduce that the molecular dynamics of single order parameter systems must obey an additional restriction, *i.e.*, the limiting case of “free volume model” must be employed. Thereby, our study reveals an important feature of the molecular dynamics of the single order parameter systems, *i.e.*, its exclusive dependence on the local density fluctuations. It suggests that the structural differences between the liquid and the glass for the hard sphere systems can be described by only one order parameter. Thus, we believe that the further studies of a dependence of order parameter on cooling or heating rates may give an answer as to whether or not another restriction for the glass transition exists, and hence some relationship among the thermodynamic coefficients takes place in this process.

## Methods

In order to calculate the isobaric exapansivity 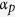 and isothermal compressibility 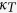, we use the approximation of the volumetric data by our new equation of state (eq. (9) in Ref. 53) for glibenclamide (GLB)^33^, verapamil hydrochlorine (VH)^54^, carvedilol base (CB), ibuprofen (IBP), indometacin (IND), N,N-dimethyl-3-methylbenzamide (DEET) (all in Ref. 55)polystyrene (PS)^56^, and polyvinylacetate (PVAc)^53^. The values of isothermal, isochoric and isobaric fragilities are estimated from temperature-volume version^40^ of the entropic Avramov model^57^, which are earlier reported for GLB, VH, CB, IND, IBP, DEET, PVAc (all in Ref. 55). The isobaric heat capacity data for GLB (also reported in Ref. 33)VH, CB, IBP, IND, and DEET have been measured at ambient pressure by using the differential scanning calorimetery with stochastic temperature modulation (TOPEM), whereas literature reports on 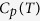 at 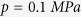 for PS^56^, PVAc^58^, have been used. It also may be of value to mention that the determination of the total entropy is not required to calculate the values of 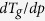 according to equation (7) since the total entropy can be expressed by the well-known thermodynamic formula, 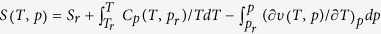, where 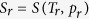 is the constant entropy of the reference state (which can be defined by the glass transition temperature at ambient pressure) and then 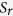 can be omitted for estimation 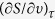.

## Additional Information

**How to cite this article**: Koperwas, K. *et al.* Thermodynamic consequences of the kinetic nature of the glass transition. *Sci. Rep.* **5**, 17782; doi: 10.1038/srep17782 (2015).

## Supplementary Material

Supplementary Information
