# Supplementary material for: Thermodynamic consequences of the kinetic nature of the glass transition
Source: Sci Rep. 2015 Dec 10;5:17782. doi: 10.1038/srep17782 (PMC4674716; doi:10.1038/srep17782)
Supplement: Supplementary Information [file srep17782-s1.pdf]

Thermodynamic consequences of the kinetic nature of the glass transition.

Kajetan Koperwas[1,2,*], Andrzej Grzybowski[1,2], Satya N. Tripathy[1,2], Elzbieta Masiewicz[1,2] & Marian Paluch[1,2]

[1]*Institute of Physics, University of Silesia, Uniwersytecka 4, 40-007 Katowice, Poland*

[2]*Silesian Center for Education and Interdisciplinary Research, 75 Pulku Piechoty 1A, 41-500 Chorzow, Poland*

*Corresponding author's email: kkoperwas@us.edu.pl

SUPPLEMENTARY INFORMATION

1. UNIVERSAL EQUATION FOR THE PRESSURE COEFFIIENT OF THE GLASS TRANSITION TEMPERATURE IN THE CONTEXT OF DENSITY SCALING OF THE TOTAL SYSTEM ENTROPY

Very recently we have shown, that in the contrast to some results based on theoretical and simulations studies[1,2,3,4,5], τ_α is not in general a single variable function of total system entropy[6]. Nevertheless, it has to be noted that obtained result seems to be in accord with the newly reformulated theory of isomorphs[7,8]. Moreover, we have found that S can be scaled versus Tv^{γ_S}, where γ_S is only material dependent. Then, if the density scaling of structural relaxation times is fulfilled, we obtained that τ_α is a function of v and S. Therefore in this

paragraph we consider dT_g / dp in the context of the dependence $\tau_\alpha(v,S)$. In this case the complete differential of the structural relaxation time $d\tau_\alpha(v,S)$, at the glass transition defined at a constant value of τ_g, equals $0 = d\tau_g(v,S) = \left(\frac{\partial\tau}{\partial v}\right)_S dv + \left(\frac{\partial\tau}{\partial S}\right)_v dS$. Since v and S depend on T and p, one can rewrite last expression in the following form $0 = \left(\frac{\partial\tau}{\partial v}\right)_S \left[\left(\frac{\partial v}{\partial T}\right)_p dT + \left(\frac{\partial v}{\partial p}\right)_T dp\right] + \left(\frac{\partial\tau}{\partial S}\right)_v \left[\left(\frac{\partial S}{\partial T}\right)_p dT + \left(\frac{\partial S}{\partial p}\right)_T dp\right]$. After regrouping and using Maxwell relations we obtain that $\frac{dT_g}{dp} = \frac{-\left[\left(\frac{\partial\tau}{\partial v}\right)_S (-v\kappa_T) + \left(\frac{\partial\tau}{\partial S}\right)_v (-v\alpha_p)\right]}{\left(\frac{\partial\tau}{\partial v}\right)_S v\alpha_p + \left(\frac{\partial\tau}{\partial S}\right)_v \frac{C_p}{T}}$. Since $\left(\frac{\partial\tau}{\partial S}\right)_v \left(\frac{\partial v}{\partial\tau}\right)_s \left(\frac{\partial S}{\partial v}\right)_\tau = -1$, the above equation transforms to $\frac{dT}{dp} = \frac{\left(\frac{\partial S}{\partial v}\right)_\tau v\kappa_T - v\alpha_p}{\left(\frac{\partial S}{\partial v}\right)_\tau v\alpha_p - \frac{C_p}{T}}$, where mentioned in the main text relation $\left(\frac{\partial S}{\partial v}\right)_\tau = \left(\frac{\partial S}{\partial v}\right)_T + \left(\frac{\partial S}{\partial T}\right)_v \left(\frac{\partial T}{\partial v}\right)_\tau$, can be used. Then, finally we obtain the equation identical with Eq. (4a) in the main text *i.e.,* with the equation received in the case of $\tau_\alpha(T,v)$. Similarly, if one considers $\tau_\alpha(p,S)$, the formula for dT_g / dp, which is the same as Eq. (4b) (and hence also as Eq. (4a)), could be obtained.

2. CONNECTION BETWEEN THERMODYNAMICS AND DYNAMICS RESULTS FROM UNIVERSAL EQUATION FOR THE PRESSURE COEFFICIENT OF THE GLASS TRANSITION TEMPERATURE

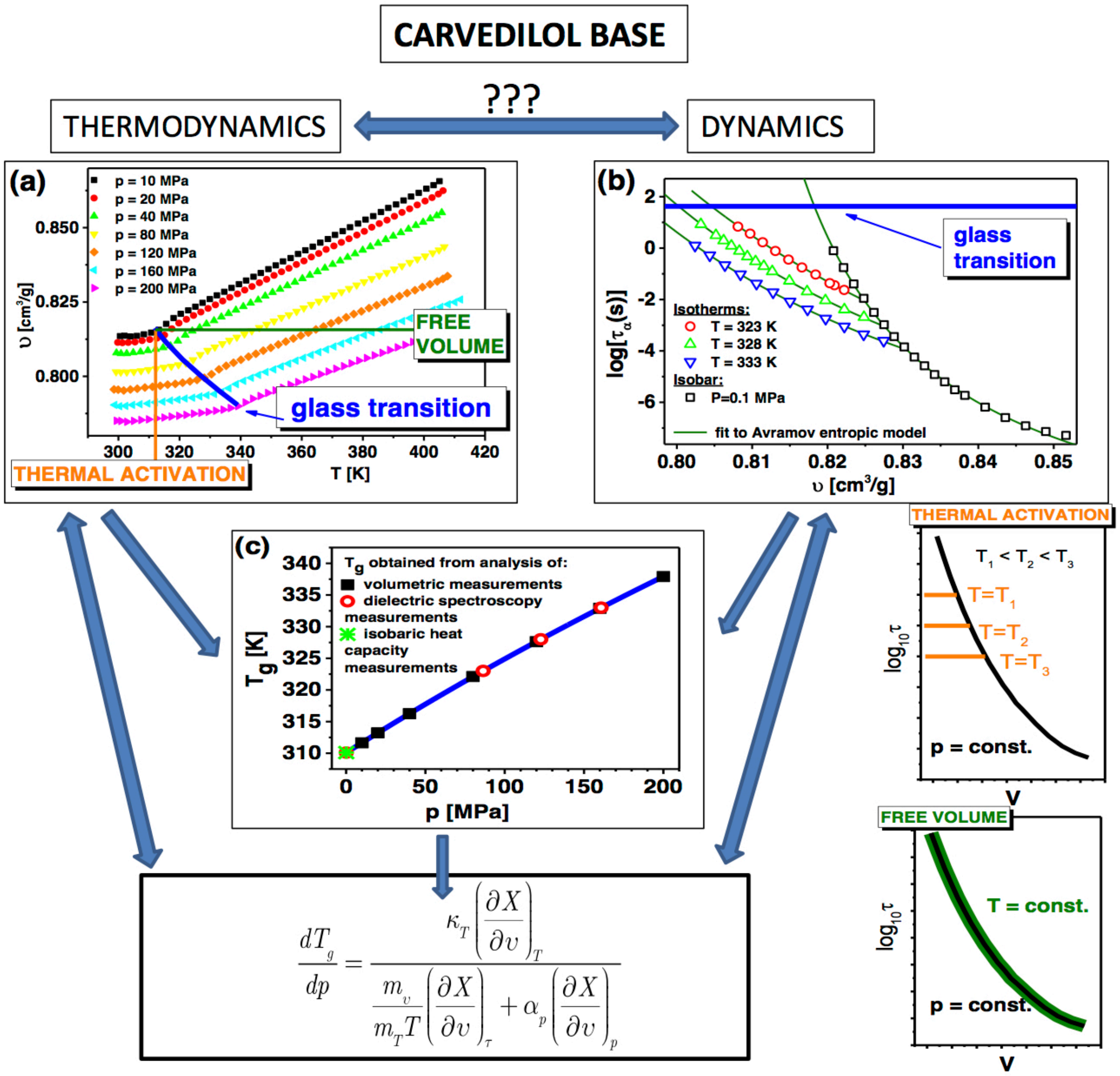

From the volumetric measurements (a) one can clearly see that the glass transition does not take place at constant volume or temperature, thus T_g depends on p. However, the glass transition line is in a perfect agreement with the isochronal conditions (c) established in dielectric spectroscopy experiment (b). Taking into account the definition of the glass transition temperature formulated on the assumption of the isochronal conditions we find a

universal equation for dT_g / dp, which is independent on the thermodynamic quantities, which changes reflect the glass formation, e.g., $X = v$ for the volumetric measurements or $X = S$ in the case of the calorimetery measurements. Moreover, our universal equation openly takes into account the roles of the temperature and the volume in the molecular dynamics, which is also reflected in the pure thermodynamic measurements, i.e., volumetric measurements. Therefore, our result fastens the thermodynamics and dynamics of supercooled liquid together, in simple way. Since, universal equation derived by us can describe the both limiting cases, i.e., "free volume" and "thermal activation", it clearly shows that dynamics of supercooled liquids is reflected in their thermodynamic in a such big way that none relation among the thermodynamic coefficients at the glass transition can be established, unless the additional restriction (or "feature" of the molecular dynamics) met at the glass transition would be found. Above conclusion opens new perspectives to the old standing open questions about the use of the equivalent to the Ehrenfest equations not only to the glass formation but also to any isochronal process.

[1] Rosenfeld, Y. A quasi-universal scaling law for atomic transport in simple fluids. *J. Phys. Cond. Matter* **11**, 5415-5427 (1999).

[2] Pont, M. J., Errington, J. R. & Truskett, T. M. Generalizing Rosenfeld's excess-entropy scaling to predict long-time diffusivity in dense fluids of Brownian particles: From hard to ultrasoft interactions. *J. Chem. Phys.* **134**, 081101-1-4 (2011).

[3] Chopra, R., Truskett, T. M. & Errington, J. R. Excess entropy scaling of dynamic quantities for fluids of dumbbell-shaped particles. *J. Chem. Phys.* **133**, 104506-1-11 (2010).

[4] Chopra, R., Truskett, T. M. & Errington, J. R. On the Use of Excess Entropy Scaling To Describe Single-Molecule and Collective Dynamic Properties of Hydrocarbon Isomer Fluids. *J. Phys. Chem. B* **114**, 16487-16493 (2010).

[5] Galliero, G., Boned, C. & Fernandez, J. Scaling of the viscosity of the Lennard-Jones chain fluid model, argon, and some normal alkanes. *J. Chem. Phys.* **134**, 064505-1-8 (2011).

[6] Grzybowska, K., Grzybowski, A., Pawlus, S., Pionteck, J. & Paluch, M. Role of entropy in the thermodynamic evolution of the time scale of molecular dynamics near the glass transition. *Phys. Rev. E* **91**, 062305-1-13 (2015).

[7] Bohling, L., Bailey, N. P., Schrøder, T. B. & Dyre, J. C. Estimating the density-scaling exponent of a monatomic liquid from its pair potential. *J. Chem. Phys.* **140**, 124510-1-7 (2014).

[8] Schrøder, T. B. & Dyre, J. C. Simplicity of condensed matter at its core: Generic definition of a Roskilde-simple system *J. Chem. Phys.* **141**, 204502-1-9 (2014).
